# The relationship between moral distress, burnout, and considering leaving a hospital job during the COVID-19 pandemic: a longitudinal survey

**DOI:** 10.1186/s12912-023-01407-5

**Published:** 2023-07-26

**Authors:** Robert G. Maunder, Natalie D. Heeney, Rebecca A. Greenberg, Lianne P. Jeffs, Lesley A. Wiesenfeld, Jennie Johnstone, Jonathan J. Hunter

**Affiliations:** 1 Mount Sinai Hospital, Toronto, Canada; 2 University of Toronto, Toronto, Canada; 3 Lunenfeld-Tanenbaum Research Institute, Toronto, Canada

**Keywords:** Moral distress, Burnout, Healthcare, COVID-19, Occupational stress

## Abstract

**Background:**

Previous research suggests that moral distress contributes to burnout in nurses and other healthcare workers. We hypothesized that burnout both contributed to moral distress and was amplified by moral distress for hospital workers in the COVID-19 pandemic. This study also aimed to test if moral distress was related to considering leaving one’s job.

**Methods:**

A cohort of 213 hospital workers completed quarterly surveys at six time-points over fifteen months that included validated measures of three dimensions of professional burnout and moral distress. Moral distress was categorized as minimal, medium, or high. Analyses using linear and ordinal regression models tested the association between burnout and other variables at Time 1 (T1), moral distress at Time 3 (T3), and burnout and considering leaving one’s job at Time 6 (T6).

**Results:**

Moral distress was highest in nurses. Job type (nurse (co-efficient 1.99, p < .001); other healthcare professional (co-efficient 1.44, p < .001); non-professional staff with close patient contact (reference group)) and burnout-depersonalization (co-efficient 0.32, p < .001) measured at T1 accounted for an estimated 45% of the variance in moral distress at T3. Moral distress at T3 predicted burnout-depersonalization (Beta = 0.34, p < .001) and burnout-emotional exhaustion (Beta = 0.38, p < .008) at T6, and was significantly associated with considering leaving one’s job or healthcare.

**Conclusion:**

Aspects of burnout that were associated with experiencing greater moral distress occurred both prior to and following moral distress, consistent with the hypotheses that burnout both amplifies moral distress and is increased by moral distress. This potential vicious circle, in addition to an association between moral distress and considering leaving one’s job, suggests that interventions for moral distress may help mitigate a workforce that is both depleted and burdened with burnout.

## Background

Moral distress is a phenomenon that was originally defined as occurring when “one knows the right thing to do, but institutional constraints make it nearly impossible to pursue the right course of action” [1]. The concept emerged from nursing scholarship [1], perhaps because frontline nurses are in a position that is particularly sensitive to institutional constraints on care, and has been largely applied within healthcare. A synthetic review of the subsequent literature which aimed to provide conceptual clarity suggested that the necessary conditions for moral distress are broader than the original definition and include the experience of a morally challenging event, the experience of psychological distress, and a direct causal relationship between the event and the distress [2]. This definition de-emphasizes the necessity of knowing the right thing to do (i.e. it acknowledges that moral distress sometimes arises in the context of moral uncertainty) and de-emphasizes the necessity of constraint as a cause; rather it shifts to focus on the experience of a threat to one’s moral integrity and self-worth.

While first identified in critical care nurses, moral distress has now been identified in several other healthcare professions [3, 4] including psychologists [5], physicians [6, 7], respiratory therapists [8], and social workers [9], although greater moral distress has been reported in nurses than in other healthcare professionals [4, 7]. Causes of healthcare workers’ moral distress can be broadly grouped into categories that include patient and family situations, individual constraints, unit or team dynamics, and the original focus of moral distress, organizational constraints [10]. Situations within these categories that have been found to commonly cause moral distress include families requesting to hide a terminal illness from a patient [11], feelings of powerlessness [12], poor communication or distrust between colleagues – particularly when it causes diminished quality of care [10, 12, 13], and access to limited resources or resource allocation [13–15].

While moral distress refers to the distress experienced at the time of an event or choice, reactive distress [1], later described as moral residue [16], refers to the long-lasting effects that persist following the event. Moral residue describes the concept of carrying the distress and unresolved feelings of a morally distressing situation forward into one’s moral life, altering one’s sense of self [16, 17]. Furthermore, repeated exposure to moral distress can result in compounding feelings of moral distress and residue, termed the ‘crescendo effect’ [18], which can lead a healthcare provider to react more strongly to a similar morally distressing situation the next time it occurs.

In a review of quantitative studies of moral distress in 2015, greater intensity of moral distress was associated with older age, longer experience, and a reported a lack of power within the healthcare system [19]. Moral distress was also reported to be greater in acute care settings [20]. Potential consequences of moral distress include burnout [21, 22], frustration and anger [23], and an intention to leave one’s job [7, 20, 24, 25]. It has been noted, in particular, that the crescendo effect resulting from repeated exposure to moral distress leads to cynicism, detachment, and reduced commitment and integrity [18].

Working conditions in healthcare during the COVID-19 pandemic increased the exposure of healthcare workers to situations of moral uncertainty (for example, situations in which healthcare workers’ personal safety and duty to care are in conflict), and situations in which societal and organization constraints prevented providing optimal care, or otherwise “doing the right thing” [26]. Additionally, COVID-19 elicited moral uncertainty when healthcare workers, who were accustomed to thinking about the best interest of their patients as individuals, were required to also consider the ethical value of utility – the greatest good for the greatest number. Many healthcare organizations were required to more explicitly consider how to prioritize needs of individual patients against societal needs. Consequently, clinicians were required to operationalize policies that were a far departure from how they typically conceptualized the provision of quality care, such as triage to determine access to limited resources. Additional pandemic-related conditions that have been reported to contribute to moral distress include limits on contact between patients and families [27][28, 29], constraints imposed by virtual care [30], and perceived deficits in executive leadership and communication [27, 31, 31]. During the pandemic, moral distress has been reported to be greater among staff who see patients, in those who are in frontline roles, and in nurses compared to support staff [29, 32].

In the COVID-19 pandemic, moral distress in hospital workers has been consistently related to negative psychological outcomes, including burnout, and symptoms of anxiety, depression, and posttraumatic stress [32–35]. The intention to leave one’s job during the pandemic has also been reported to increase with greater moral distress [34, 36], a consequence that has great systemic significance in an era in which understaffing is widely reported.

Most pandemic-era studies reporting on potential contributors to moral distress and its potential consequences have been cross-sectional surveys. In these studies, it is not possible to determine if psychological distress and burnout, which are conceptualized as consequences of moral distress, might also serve as contributors to or amplifiers of moral distress, for example by depleting internal resources that are needed to cope with morally challenging circumstances. Two longitudinal studies reported that moral distress predicted subsequent burnout but did not test causation in the opposite direction [35, 37]. Thus, there is a lack of longitudinal research which assesses potential (i) contributors to moral distress, including dimensions of burnout and psychological distress, measured prior to moral distress and (ii) consequences of moral distress at a subsequent time. The current study aims to fill this gap using data collected in a longitudinal study of a cohort of hospital workers who completed surveys approximately quarterly over six time-points between September 2020 and February 2022 during the COVID-19 pandemic. The surveys included measures of moral distress, burnout, psychological distress, as well as self-efficacy and resilience characteristics, which are possible buffers of moral distress.

## Methods

### Aim

This study aimed to assess contributors to moral distress and consequences of moral distress at a subsequent time in healthcare workers during the COVID-19 pandemic.

### Design

This study is a secondary analysis of the results of a prospective longitudinal survey of a cohort of hospital workers, with data collected approximately quarterly over six time-points between September 2020 and February 2022.

### Setting

The study was conducted at an urban hospital teaching hospital with two sites, an acute care general hospital and a rehabilitation hospital, in Toronto, Canada.

### Participants

Participation was open to hospital staff of all occupations (professional and non-professional, employees, physicians, retail employees, and contractors), learners, and volunteers. Staff were informed of the survey via emails from the organization to all staff or by their chiefs and directors, and by posters in high traffic areas of the hospital. Once the cohort was established at the first survey (T1), the same participants were invited back for each subsequent survey wave.

### Survey Methods

Detailed survey methods have been described previously [38]. An online survey of occupational and psychological characteristics of participants was conducted at six time-points. The first survey (T1) occurred from Sept 21-Nov 15, 2020. Of 884 respondents who provided consent in a pre-survey recruitment phase, 538 (61.0%) completed a T1 survey formed the cohort invited to subsequent survey waves. Subsequent surveys were conducted in these time periods: Jan 25-Feb 15, 2021 (T2), Apr 26-May 16, 2021 (T3), Jul 26-Aug 15, 2021 (T4), Oct 25–Nov 14, 2021 (T5), and Jan 24-Feb 13, 2022 (T6). All surveys were completed online using software that is compliant with local privacy standards (Alchemer, Louiseville, CO).

Participants were randomized 1:1 to a shorter or longer version of the survey, using an online randomizer in blocks of eight. Since the moral distress measure was only included in the longer version, this study only includes the 50% of participants who were randomly assigned to the longer survey. Among the 281 participants who completed the longer version of the survey at T1, the participation rate at subsequent time points (numerator calculated as the number of surveys returned that included a valid measure of emotional exhaustion, psychological distress, or both) was: T2 N = 255 (91%), T3 N = 218 (78%), T4 N = 210 (75%), T5 N = 203 (72%), T6 N = 192 (68%). A gift card (about US$15 value) was provided for each completed survey.

### Measures

The surveys included measures of moral distress, burnout, psychological distress, as well as self-efficacy and resilience characteristics, which are possible buffers of moral distress.

Burnout was measured with the Maslach Burnout Inventory (MBI-HSS), which measures the frequency of experiences related to emotional exhaustion (9 items), depersonalization (5 items), and diminished personal accomplishment (8 items) on a 7-point Likert scale from “never” to “every day” [39]. This analysis included burnout scores measured at T1, T3 and T6. Cronbach’s alpha at these time points varied from 0.94 to 0.96 (emotional exhaustion), 0.87-0.92 (depersonalization), and 0.83-0.91 (personal accomplishment).

Most studies of moral distress, but not all [23, 40], have used versions of the Moral Distress Scale[12, 41, 42] which measures the frequency of relevant events, the intensity of related moral distress, and their product (frequency X intensity). We used a version healthcare providers, the Measure of Moral Distress for Healthcare Providers (MMD-HP), which surveys 27 potential contributors to moral distress and yields a total score which is the product of frequency (scored 0 “never” to 4 “frequently”) times severity of distress (scored 0 “none” to 4 “very distressing”) for each item. The product of these two measures (each 0 to 4) ranges from 0 to 16 [41]. In this current study, moral distress scores were non-parametrically distributed and highly skewed toward zero. Given this distribution, continuous scores were transformed to an ordinal variable in which each category included one third of participants (minimal = 0–5; moderate 6–64, high 65–432), as has been done previously [7].

Psychological distress is comprised of depressive and anxiety symptoms. Psychological distress was measured with the Kessler K6, which has 6 items scored from 0 to 4, yielding a range of 0–24. The K6 strongly discriminates between community cases and non-cases of psychiatric disorders and has appropriate sensitivity and specificity [43, 44]. This analysis used psychological distress measured at T1. Cronbach’s alpha was 0.85.

Resilience characteristics were measured with the Resilience at Work scale [45]. Subscales measure “living authentically” (3 items), “finding your calling” (4 items), “maintaining perspective” (3 items), “managing stress” (4 items), “interacting cooperatively” (2 items), “staying healthy” (2 items), and “building networks” (2 items). Each item is scored on a 7-point Likert scale (0–6). We used the overall sum of all items measured at T1 for analyses. Cronbach’s alpha was 0.87.

Pandemic self-efficacy was measured with an instrument first developed for the 2009 H1N1 pandemic [46]. It has 23 items probing confidence in one’s ability to meet pandemic-related challenges (e.g. ‘trust in the infection control procedures that are in place’, ‘perform duties that are outside your usual job’) scored on a 5-point scale [1–5], yielding a score from 23 to 115. Pandemic self-efficacy at T1 was included in this analysis. Cronbach’s alpha was 0.93.

Consideration of leaving one’s job was measured at T6 with two questions answered yes or no, “Are you considering leaving your current job?” and if answered yes, “Are you considering leaving healthcare?” Responses were collapsed into a three-category variable: not considering leaving job; considering leaving current job, but not healthcare; considering leaving healthcare.

### Analysis

At T1, participants were sorted into four categories of occupational role. The first category was comprised of nurses and nursing students. The second was comprised of other staff with professional qualifications (i.e. who are regulated by a professional college, or equivalent) and their students. The third and fourth categories were non-professional staff or volunteers with or without close patient contact, respectively. Close patient contact was defined as being within two metres of a patient for more than 15 min in the previous month.

Descriptive statistics were used to characterize demographic variables. These were compared between occupational groups using Chi-squared tests. We planned to exclude staff without patient contact from the analysis if the expectation that the concept of moral distress typically doesn’t apply to these roles was confirmed by very low scores. To explore the types of situations most frequently associated with medium or high moral distress, items on the moral distress scale were ranked by the proportion of participants reporting a score in the upper two terciles for that item.

We tested the association between moral distress at T3 and potential contributors to moral distress measured at T1 (three dimensions of burnout, psychological distress, pandemic self-efficacy, resilience characteristics) using ordinal regression. Age and job type were included in this analysis a covariates because prior research identifies them as predictors [4, 7, 19].

Moral distress at T3 was tested as a predictor of four T6 variables: the three dimensions of burnout, using a linear regression model for each potential outcome, and consideration of leaving one’s job, using ordinal regression. Again age, and job type were entered as covariates. In linear regression, job type was collapsed to a binary variable: nurses (occupational category 1) vs. all others.

Analyses were conducted using IBM SPSS Statistics 28 (Armonk, New York). Significance was set at p < .05 (double-sided).

## Results

Moral distress was measured in 213 survey participants (46 nurses, 71 other healthcare professionals, 34 non-professional staff with close patient contact, and 62 non-professional staff without close patient contact). The characteristics of these participants, by job type, are presented in Table 1.

**Table 1:** Demographic characteristics and moral distress by job type

		Nurse		Other Health-care Professional		Non-professional with Patient Contact		Non-professional with no Patient Contact			
		**N = 46**		** N = 71**		** N = 34**		** N = 62**			
		**n**	**%**	**n**	**%**	**n**	**%**	**n**	**%**	**Chi** ^**2**^	**p**
**Age**	**18–30**	19	41	18	25	10	29	18	29		
	**31–40**	12	26	21	30	11	32	14	23		
	**41–50**	10	22	18	25	7	21	15	24		
	**Over 50**	5	11	14	20	8	18	15	24	6.3	0.71
**Gender**	**Female**	44	96	56	79	28	82	49	79		
	**Other**	2	4	15	21	6	18	13	21	6.8	0.08
**Education**	**Graduate or professional**	15	33	64	90	15	44	28	45		
	**Other**	31	67	7	10	19	56	34	55	48.9	< 0.001
**Marital**	**Married or Common-law**	27	59	44	62	20	59	32	52		
	**Single**	19	41	24	34	13	38	24	39		
	**Separated, divorced, widowed**	0	0	3	4	1	3	6	10	6.8	0.34
Moral distress*	**Minimal**	2	4	9	13	11	32	49	79		
	**Medium**	16	35	29	41	17	50	9	15		
	**High**	28	61	33	47	6	18	4	7	100.3	< 0.001

* Moral distress measured with the Measure of Moral Distress for Healthcare Providers. Range of scores: minimal = 0–5; moderate 6–64, high 65–432

There was significant difference in education by job type. There was a large difference in moral distress scores by job category (Chi^2^ = 100.3, p < .001) with 79% of participants who had jobs not involving regular patient contact scoring in the range of minimal moral distress. This confirmed the expectation that moral distress is most relevant in patient-facing hospital roles, and so the remainder of the analysis was limited to the 151 participants in patient-facing jobs. The events that were most frequently endorsed as contributing to moral distress at a medium or high level are listed in Table 2.

**Table 2:** Sources of moral distress with median score greater than zero

Experience	Median*	Mean
Experience compromised patient care due to lack of resources/equipment/bed capacity.	3	4.2
Witness low quality of patient care due to poor team communication.	3	3.8
Follow the family’s insistence to continue aggressive treatment even though I believe it is not in the best interest of the patient.	3	3.7
Be required to care for more patients than I can safely care for.	2	3.8
Watch patient care suffer because of a lack of provider continuity.	2	3.4
Have excessive documentation requirements that compromise patient care.	1	3.7
Experience lack of administrative action or support for a problem that is compromising patient care.	1	3.3
Fear retribution if I speak up.	1	3.2
Feel pressured to order or carry out orders for what I consider to be unnecessary or inappropriate tests and treatments.	1	2.8
Participate on a team that gives inconsistent messages to a patient/family.	1	2.8
Be unable to provide optimal care due to pressures from administrators or insurers to reduce costs.	1	2.8
Be required to care for patients who have unclear or inconsistent treatment plans or who lack goals of care.	1	2.8
Be required to work with abusive patients/family members who are compromising quality of care.	1	2.8
Be required to work with other healthcare team members who are not as competent as patient care requires.	1	2.4
Witness healthcare providers giving “false hope” to a patient or family.	1	2.2

* Median and mean of item scores on Measure of Moral Distress for Healthcare Providers. Possible item scores range from 0 to 16

Variables measured at T1 were tested as predictors of medium or high moral distress measured at T3 (Table 3). Depersonalization and job type were significantly associated with higher moral distress. There was no further significant contribution from emotional exhaustion, diminished personal accomplishment, psychological distress, pandemic self-efficacy, or resilience characteristics. This full model (Table 3 A) was estimated to account for 47% of the variance in moral distress. In the final model (Table 3B), non-significant predictor variables were removed to improve the precision of the parameter estimates for the main effect terms. This model, which included just depersonalization and job category, was estimated to account for 45% of the variance in moral distress.

**Table 3:** Predictors measured in Fall, 2020 (T1) of Moral Distress measured in Spring 2021 (T3) as determined by ordinal regression

Predictors of Moral Distress at T3	Estimate	SE	df	Sig.	Nagelkerke Pseudo R2
**A. Full model**					
T1 Burnout - emotional exhaustion	0.008	0.021	1	0.71	0.47
T1 Burnout - depersonalization	0.241	0.065	1	< 0.001	
T1 Burnout - personal satisfaction	0.003	0.026	1	0.91	
T1 Psychological distress	0.071	0.060	1	0.24	
T1 Pandemic self-efficacy	− 0.019	0.019	1	0.33	
T1 Resilience characteristics	0.006	0.025	1	0.81	
Age	− 0.011	0.018	1	0.51	
Job category = Nurse	1.982	0.547	1	< 0.001	
Job category = Other Healthcare prof.	1.752	0.475	1	< 0.001	
Job category = Non-prof. with patient contact	0		0		
**B. Final model**					
T1 Burnout - depersonalization	0.315	0.058	1	< 0.001	0.45
Job category = Nurse	1.985	0.519	1	< 0.001	
Job category = Other Healthcare prof.	1.443	0.445	1	< 0.001	
Job category = Non-prof. with patient contact	0		0		
**C. Exploratory model**					
Pre-existing depersonalization	2.044	0.387	1	< 0.001	0.47
Concurrent depersonalization	0.223	0.237	1	0.35	
Job category = Nurse	1.843	0.539	1	< 0.001	
Job category = Other Healthcare prof.	1.426	0.477	0	0.003	
Job category = Non-prof. with patient contact	0		0		

In order to try to distinguish the effects of concurrent depersonalization from the effects of pre-existing depersonalization, T1 depersonalization was regressed on T3 depersonalization in a linear regression with the predicted variance and residual variance saved as new variables, representing variance in T1 depersonalization controlling for T3 (“pre-existing”) and variance in T3 depersonalization controlling for T1 (“concurrent”), respectively. An exploratory regression analysis to explain moral distress category was then conducted (Table 3 C) including three predictor variables: job type, pre-existing depersonalization, and concurrent depersonalization. This analysis found a significant contribution of pre-existing depersonalization and of job category, and found no significant contribution from the concurrent depersonalization. The exploratory model was estimated to account for 47% of the variance in moral distress. Results of this analysis did not differ materially when the regression was conducted using log-transformed variables.

Moral distress at T3 was tested as a predictor of each of the three dimensions of burnout measured at T6. The results (Table 4), show a significant contribution of moral distress to subsequent emotional exhaustion and depersonalization, controlling for age and job category. A weaker trend towards moral distress predicting subsequent diminished personal accomplishment failed to reach significance (p = .06).

**Table 4:** Moral distress, age, and job category as predictors of subsequent (T6) burnout as determined by linear regression

	Beta	Signif.	R^2^
**A. Predicting T6 Burnout-emotional exhaustion**			0.24
T3 Moral distress	0.38	< 0.001	
Nurse (Y/N)	0.22	0.007	
Age	− 0.03	0.72	
**B. Predicting T6 Burnout-depersonalization**			0.22
T3 Moral distress	0.34	< 0.001	
Nurse (Y/N)	0.14	0.09	
Age	− 0.19	0.03	
** C. Predicting T6 Burnout-personal accomplishment**			0.13
T3 Moral distress	− 0.18	0.06	
Nurse (Y/N)	− 0.21	0.03	
Age	0.15	0.11	

The distribution of responses to the question about considering leaving one’s job, asked at T6 was: not considering leaving N = 87, 68.5%; considering leaving current job but not healthcare N = 20, 15.7%; considering leaving healthcare N = 20, 15.7% (24 participants did not complete the T6 survey). When prior (T3) moral distress was tested as a predictor of consideration of leaving one’s job, it was significant (Table 5), controlling for emotional exhaustion, depersonalization, age, and job category. The model was estimated to account for 20% of the variance in this consideration.

**Table 5:** Predictors of considering leaving one’s job as determined by ordinal regression

Predictors of considering leaving one’s job	Estimate	SE	df	Sig	Nagelkerke Pseudo R^2^
T3 Emotional exhaustion	0.029	0.022	1	0.19	0.20
T3 Depersonalization	− 0.028	0.039	1	0.47	
T3 Moral distress = minimal	-2.646	1.201	1	0.03	
T3 Moral distress = medium	− 0.414	0.508	1	0.42	
T3 Moral distress = high	0		0		
Age	− 0.024	0.022	1	0.29	
Job category = Nurse	-1.073	0.598	1	0.073	
Job category = Other healthcare prof.	-1.720	0.588	1	0.003	
Job category = Non-prof. with patient contact	0		0		

To illustrate the strength of the relationship between moral distress and considering leaving one’s job, we calculated the proportion of participants endorsing each of the job-leaving responses among those previously reporting minimal, medium or high moral distress. This distribution revealed that compared to participants reporting minimal moral distress (considering leaving current job 5%; considering leaving healthcare 0%), considering leaving work was more common in those reporting medium and high moral distress (leaving current job 15% and 21% respectively; leaving healthcare 20% and 17% respectively; linear by linear association = 4.0, p = .046).

## Discussion

This study of hospital-based healthcare workers conducted during the COVID-19 pandemic documents the frequency of experiencing medium or high moral distress and the type of events that commonly contributed to this experience. The longitudinal study design facilitated determining that aspects of burnout that were associated with moral distress occurred both prior to (Table 3) and following (Table 4) moral distress, consistent with the hypotheses that burnout both amplifies moral distress and occurs because of moral distress. Statistically parsing pre-existing and concurrent aspects of burnout (specifically its depersonalization dimension) suggested that it is the pre-existing component that is more strongly associated with experiencing more severe moral distress, which supports the association being due a causal relationship between these phenomena.

Moral distress was strongly related to job type, as illustrated by the finding that high moral distress was reported 61% of nurses, 47% of other healthcare professionals, 18% of non-professionals in patient-facing jobs, and only 7% of non-professionals in non-patient facing roles. The greater impact of moral distress on nurses than on other healthcare professionals is consistent with pre-pandemic research [4, 7], although importantly moral distress is not unique to nurses and so resources to mitigate this exposure should be available to all patient-facing staff. Greater moral distress in nurses than physicians has been attributed to nurses having the responsibility to carry out a care plan that they have had little input in creating [7]. Other differences between occupational roles that could contribute to differences in moral distress relate to amount of face-to-face contact with and responsibility for patients, and the differential impact of pandemic-related stresses on different occupations (for example redeployment to unfamiliar tasks, or exposure to the consequences of poor team communication or discontinuities in care).

Several of the events which were endorsed as stronger and more frequent contributors to moral distress were consistent with anecdotal observations of the nature of how hospital-based care changed during the first two years of the pandemic with optimal care and access compromised by high patient loads and a lack of sufficient resources and capacity, disruptions in the continuity of care, and impaired team functioning.

These attributions align with qualitative themes that emerged from narrative comments provided by survey participants that elucidated the spectrum, magnitude, and severity of moral distress due to stress associated with not being able to provide optimal care, feeling devalued and invisible, and incivility that was experienced by the healthcare workforce (publication pending).

The longitudinal design of the survey allowed an examination of both antecedents and consequences of moral distress. The results supported a bidirectional relationship between burnout and moral distress. Other studies report that moral distress contributes to experiencing burnout [35, 37]. The current study indicated that burnout is consistent with the interpretation that moral distress also contributes to experiencing moral distress, perhaps because a depletion of internal psychological resources makes it more difficult to tolerate or respond effectively to circumstance of moral strain or uncertainty. A causal association in this latter direction has not been reported previously to the best of our knowledge. An important limitation on this interpretation of our results is that although moral distress was only measured at T3, the experience of moral distress reported at that time may have been present both prior to and after the measurement point, as continuing moral residue, especially in an occupational context in which repeated morally challenging events were occurring for many healthcare workers. To the extent that both burnout and moral distress are continuously co-occurring, temporal sequences suggested by measuring them at different times may be spurious. Further research is needed because our longitudinal observation design can detect temporal associations that are consistent with causal relationships but cannot demonstrate causality.

This study found that the depersonalization dimension of burnout was specifically significant as an antecedent of moral distress. It is not known why depersonalization would contribute to moral distress as opposed to emotional exhaustion, which is a more direct indicator of depleted internal resources. Depersonalization (a decrement in empathy and caring about patients) can be understood as a *failure* of compassion in the face of overwhelming strain, or as a *defense strategy* in which compassionate engagement is reduced to reduce vicarious harm. Its possible contribution to moral distress is consistent with viewing depersonalization as a defense, since in principle, experiencing moral distress is contingent on caring about doing one’s best for patients’ well-being. Empirically, high levels of depersonalization are reported much less frequently than high levels of emotional exhaustion (the distribution of scores is skewed towards zero, as opposed to emotional exhaustion which is more nearly normally distributed – data not shown). Thus, the reason that depersonalization is specifically associated with moral distress may be that it may represent a later stage of more severe burnout in the context of the extraordinary stress. A previous analysis of results from the first four survey time-points supports the view that depersonalization results from the accumulative impact of occupational stress, as it demonstrated that while emotional exhaustion rose and fell over time, roughly corresponding to the waves of the pandemic case rate, depersonalization rose continuously (mean scores of 4.80, 5.32, 6.42, and 6.67 at T1 to T4 respectively [47]). This trend has continued at subsequent time points (mean score of 6.70 and 7.45 at T5 and T6).

While the finding of an association of moral distress with consideration of leaving one’s job or leaving healthcare altogether cannot be assumed to result from a causal relationship, that interpretation is logically sound: if one’s work challenges one’s sense of moral integrity sufficiently, it makes sense that one would consider looking for other work. Although considering leaving one’s job need not lead to actually making this change, in the context of the pandemic, which has led to widely reported staff shortages, this possibility is particularly important, especially if moral distress is contributing to this consideration independently of other long-standing concerns (e.g. low pay and long hours).This finding, which is consistent with previous research [7, 20, 24, 25], emphasizes the potential value of workplace interventions to reduce moral distress, in order to provide relief that does not depend on leaving the workplace. Interventions to mitigate moral distress that have been evaluated to date have focussed on education, facilitated discussion, specialist consultation services, multidisciplinary rounds, self-reflection and narrative writing, although more robust evaluative studies are needed [48].

This longitudinal survey study with multiple repeated measures in the same cohort is an advance on previous studies of the relationship between moral distress burnout and considering leaving one’s healthcare job. However, this study also has important limitations. As discussed above, moral distress may be a persistent state, described as moral residue. As a result, measuring moral distress at T3 does not assure that what is being measured follows the variables measured at T1, and precedes the variables measured at T6, *as experienced by the participant*. This concern could be addressed in future research with repeated measures of moral distress to determine its changes over time. This study took place at two sites of a single hospital organization, although the phenomena that it studies are expected to occur widely; replication in other settings would be valuable. The method of recruiting participants into the cohort was not a sampling strategy that would ensure a representative sample. Finally, while retention of participants in repeated surveys while working under extraordinarily stressful conditions was good under the circumstances (68% retention through to T6), it is possible that the differences in the characteristics of those who dropped out over time or persisted biased the results.

In conclusion, this study suggests that elements of burnout are both antecedents to moral distress and consequences of moral distress, which could cause a vicious cycle for hospital workers during a pandemic. An association between moral distress and considering leaving one’s job emphasizes the importance for healthcare organizations of mitigating the impact of this experience as much as possible in order to maintain an effective workforce. Moral distress potentially occurs in any hospital workers who have sustained patient contact but is especially pronounced among nurses, giving rise to the potential for a workforce which is both depleted and burdened with burnout.

## Conclusions

Moral distress among hospital workers was strongly related to job type and highest in nurses. Aspects of burnout both contribute to moral distress and are increased by moral distress, a bidirectional relationship which could lead to a vicious circle. The depersonalization dimension of burnout may be especially significant as an antecedent of moral distress. Experiencing higher levels of moral distress is associated with considering leaving one’s job or leaving healthcare. These results indicate that reducing moral distress may be an important goal for healthcare workers in order to reduce burnout and maintain an effective workforce.

## Data Availability

The datasets used and analysed during the current study are available from the corresponding author on reasonable request.
